# Distinguishing malaria and influenza: Early clinical features in controlled human experimental infection studies^☆^

**DOI:** 10.1016/j.tmaid.2012.03.008

**Published:** 2012-07

**Authors:** Patrick J. Lillie, Christopher J.A. Duncan, Susanne H. Sheehy, Joel Meyer, Geraldine A. O'Hara, Sarah C. Gilbert, Adrian V.S. Hill

**Affiliations:** a Centre for Clinical Vaccinology and Tropical Medicine, Churchill Hospital, Oxford OX3 7LJ, UK; b The Jenner Institute Laboratories, Old Road Campus Research Building, Roosevelt Drive, Oxford OX3 7DQ, UK

**Keywords:** Experimental infection, Pandemic influenza, Predictive value, Clinical features, Healthy volunteers

## Abstract

During the H1N1 influenza pandemic (pH1N1/09) diagnostic algorithms were developed to guide antiviral provision. However febrile illnesses are notoriously difficult to distinguish clinically. Recent evidence highlights the importance of incorporating travel history into diagnostic algorithms to prevent the catastrophic misdiagnosis of life-threatening infections such as malaria.

We applied retrospectively the UK pH1N1/09 case definition to a unique cohort of healthy adult volunteers exposed to *Plasmodium falciparum* malaria or influenza to assess the predictive value of this case definition, and to explore the distinguishing clinical features of early phase infection with these pathogens under experimental conditions.

For influenza exposure the positive predictive value of the pH1N1/09 case definition was only 0.38 (95% CI: 0.06–0.60), with a negative predictive value of 0.27 (95% CI: 0.02–0.51). Interestingly, 8/11 symptomatic malaria-infected adults would have been inappropriately classified with influenza by the pH1N1/09 case definition, while 5/8 symptomatic influenza-exposed volunteers would have been classified without influenza (*P* = 0.18 Fisher's exact). Cough (*P* = 0.005) and nasal symptoms (*P* = 0.001) were the only clinical features that distinguished influenza-exposed from malaria-exposed volunteers.

An open mind regarding the clinical cause of undifferentiated febrile illness, particularly in the absence of upper respiratory tract symptoms, remains important even during influenza pandemic settings. These data support incorporating travel history into pandemic algorithms.

## Introduction

With the advent of the first pandemic of influenza of the 21st century, there was significant concern about the potential impact on healthcare infrastructure of managing pandemic H1N1 (pH1N1) infections in traditional settings. In July 2009, due to sustained community transmission of pH1N1 (with 80,000–100,000 symptomatic cases/week in England^1^), control efforts shifted to mass treatment strategies, and the UK National Pandemic Flu Service began to offer treatment of symptomatic individuals identified by telephone or internet-based triage in England.^2^ However the initial pandemic influenza triage algorithm omitted questions about travel to a malaria endemic area.^3^ As a result, at least 3 cases of malaria infection in travellers that were misdiagnosed with influenza were reported.^4,5^ While in a pandemic setting such triage may be essential for efficient recognition and management of cases, the UK case definition has been subsequently shown to be poorly predictive.^2^

In the course of separate candidate vaccine efficacy trials carried out by our unit (www.clinicaltrials.gov NCT00890760/NCT00993083), we independently exposed cohorts of unimmunised healthy control volunteers to pathogenic strains of either *Plasmodium falciparum* (*P. falciparum*) malaria or influenza A under controlled experimental conditions, and followed them closely in the early phases of infection until clinical diagnosis. These control volunteers were enrolled to ensure the reliability of the respective experimental infections and therefore did not receive any immunisations. Although these studies were conducted separately, assessment of clinical symptoms was conducted according to uniform criteria. These data afford us a unique opportunity to perform a retrospective comparison of the early clinical features that may be of use in differentiating clinically between an often uncomplicated illness in influenza and a potentially life-threatening infection in malaria, and to assess whether the clinical features included in the UK pandemic case definition alone were sufficiently discriminatory.

## Methods

### Controlled human malaria infection (CHMI)

Recruitment occurred at the Centre for Clinical Vaccinology and Tropical Medicine, Oxford, with the challenge procedure performed, using five infectious bites from *P. falciparum* 3D7-strain infected mosquitoes, at Imperial College, London. We recruited healthy malaria naïve adults aged 18–50 years old from the Oxford area. Enrolled control volunteers (*n* = 12) were seronegative for HIV, Hepatitis B virus and Hepatitis C virus and were used to assess the infectibility of the challenge inoculum. Routine haematological and biochemical tests on enrolled control volunteers were all within normal limits. These control volunteers underwent full clinical examination and safety blood tests the day prior to sporozoite challenge. All volunteers remained outpatients throughout the challenge procedure. Volunteers attended twice daily for clinical assessment as previously described.^6^ Self-reported solicited and unsolicited symptoms and routine observations (BP, pulse, temperature) were collected twice daily from day 6.5 post-challenge and guided physical examination carried out as indicated by symptoms. Thick blood films were examined twice daily for malaria parasites by blinded microscopists together with a concurrent highly sensitive quantitative polymerase chain reaction (qPCR) assay for *P. falciparum*. Treatment with a standard oral dose of artemether/lumefantrine for 3 days was administered on the detection of a single parasite by microscopy, or on the occurrence of significant symptoms + sequential positive qPCR (if blood films were negative). Volunteers were followed up daily until two consecutive negative malaria films were observed, and returned for full clinical assessment including safety blood tests on days 35, 90 and 140 post-challenge.

### Controlled human influenza infection

Recruitment occurred at the Centre for Clinical Vaccinology and Tropical Medicine, Oxford and the Wellcome Trust Clinical Research Facility, Southampton. Volunteers aged 18–45 years were initially screened by haemagglutination inhibition (HI) assay against the virus to be used in the challenge phase of the study to ensure susceptibility to challenge. Those with a titre ≤1:10 were eligible for further detailed screening. Enrolled volunteers (*n* = 12) were seronegative for HIV, Hepatitis B virus and Hepatitis C virus and had not received seasonal influenza vaccination for at least one year prior to enrolment. Routine haematological and biochemical tests on enrolled control volunteers were all within normal limits. Control volunteers underwent clinical examination, spirometry, safety blood tests and electrocardiography on entry to the quarantine facility. Two days after entry to quarantine (to allow time for observation of any symptoms of respiratory virus infections) control volunteers were challenged with intranasal administration of H3N2 influenza (A/Wisconsin/67/2005) at a dose of 1 ml of 10^5.25^ TCID50/ml. All volunteers were inoculated during the same 2-h period. After challenge, volunteers were followed up in the quarantine facility as previously described.^7^ Self-reported symptoms were collected twice daily and a physical examination by a blinded physician was carried out daily. Nasal lavage fluid for quantification of viral shedding was obtained daily. Symptoms and physician elicited clinical signs were recorded using a standardised modified Jackson scoring system^8^ (which assigns severity of symptoms such as cough, rhinorrhoea etc., on a scale of 0–3, where no symptoms = 0; just noticeable = 1; bothersome but can still do activities = 2; and bothersome and cannot do daily activities = 3) with a score of ≥4 indicating influenza disease. Safety blood tests and further spirometry and electrocardiography were performed on all volunteers whilst in quarantine. A five-day course of oseltamivir was commenced from day 5 post-challenge for all volunteers with medication provided to complete the course after discharge. Volunteers were released from quarantine on the 7th day post-challenge after a negative rapid antigen test for influenza on nasal washings was obtained. After discharge all volunteers were followed up on days 35, 91 and 181 –post-challenge for safety blood tests.

### Ethics

Clinical trial protocols (www.clinicaltrials.gov identifiers: NCT00890760 and NCT00993083) were approved by the UK Medicines and Healthcare Products Regulatory Agency and the Oxfordshire NHS Research Ethics Committee. All volunteers provided signed informed consent prior to any study procedure, and all studies were conducted according to the principles of Good Clinical Practice and the Declaration of Helsinki.

### Case definition

We examined the UK 2009 pandemic H1N1 influenza A case definition (pH1N1/09) which was used from 2nd July 2009 to determine provision of antiviral therapy in the absence of assessment by health professional. This was as follows:

• Fever ≥38 °C or history of fever,

and two or more of the following:

• Cough, sore throat, headache, rhinorrhoea, limb or joint pain.^2,9^

### Statistical analysis

We calculated the ability of the UK pH1N1/09 case definition to distinguish influenza-exposed unvaccinated volunteers from malaria-exposed unvaccinated volunteers by calculating positive and negative predictive values. We also analysed the significance of differences between proportions of early symptoms in the group of malaria-exposed and influenza-exposed volunteers, and repeated this analysis for those individuals with laboratory-confirmed influenza. Continuous variables were assessed for normality and significance of differences between central tendencies (mean or median) assessed by appropriate parametric or non-parametric tests (Student's *t* test or Mann–Whitney *U* test respectively). All statistical analysis was performed using Prism 5.0 (GraphPad), with two-tailed tests and an alpha value of <0.05 considered statistically significant.

## Results

### Participants

1/12 influenza unvaccinated control volunteers developed an asymptomatic rise in HI titre and this volunteer was excluded prior to challenge; therefore 11 unvaccinated controls were exposed to influenza infection. 11/12 malaria unvaccinated control volunteers and 8/11 influenza control volunteers subsequently developed symptoms of infection and were included in the analysis. No serious adverse events occurred in volunteers in either challenge. Demographic details of these participants are summarised in Table 1. All malaria volunteers were diagnosed on the basis of positive blood film microscopy (geometric mean parasitaemia 4030p/mL by qPCR) for *P. falciparum* a mean of 11.8 days post-challenge (Ewer K. *et al.*, manuscript submitted). 5/8 symptomatic influenza volunteers developed laboratory-confirmed influenza infection, defined as positive viral culture for challenge virus on nasal lavage fluid a mean of 2.3 days post-challenge (Lillie P.J. *et al.*, Clinical Infectious Disease *in press*).

### Predictive value of case definition

For influenza exposure the positive predictive value (PPV) of the pH1N1/09 case definition was only 0.38 (95% CI: 0.06–0.60), with a negative predictive value (NPV) of 0.27 (95% CI: 0.02–0.51). Interestingly, 8/11 symptomatic malaria-infected adults would have been inappropriately classified with influenza based on the pH1N1/09 case definition, whilst for influenza only 3/8 symptomatic (*P* = 0.18 Fisher's exact) and 3/5 volunteers (*P* = 1.0 Fisher's exact) with laboratory-confirmed influenza would have been correctly identified.

### Clinical features

Clinical features of the exposed volunteers in the influenza and malaria cohorts are summarised in Table 2. Only the incidence of cough (*P* = 0.005 Fisher's exact) and rhinorrhoea (*P* = 0.001 Fisher's exact) were significantly different between the groups. In line with this increase in upper respiratory tract symptoms in influenza-exposed volunteers, there was also a trend towards an increased frequency of sore throat (*P* = 0.06 Fisher's exact). Restricting analysis to the 5/8 symptomatic influenza-exposed volunteers with viral culture-confirmed infection, the significant increase in cough (*P* = 0.003 Fisher's exact) and rhinorrhoea (*P* = 0.001 Fisher's exact) remained, whilst the trend for sore throat became significant (*P* = 0.02 Fisher's exact). Physiological parameters did not distinguish the malaria and influenza cohorts. There were no significant differences in the peak mean heart rate (malaria 92.2 beats per minute [95% CI: 82.6–101.8], influenza 82.7 [95% CI: 69.5–96.0], *P* = 0.19 *t* test) or temperature (malaria 37.2 °C [95% CI: 36.6–37.7], influenza 37.3 [95% CI: 36.9–37.7], *P* = 0.72 *t* test) between the groups, and there were no volunteers with clinically significant hypotension in either group.

### Laboratory features

Laboratory analysis was performed at different time-points post-challenge for influenza and malaria-infected volunteers, prohibiting direct comparisons. However, grade 1 thrombocytopaenia and leucopaenia occurred with greater frequency in malaria-exposed volunteers. The diagnosis of malaria was confirmed by thick film microscopy in 12/12 volunteers, all of whom were also positive for *P. falciparum* by qPCR. The diagnosis of influenza was confirmed by viral culture in 5/8 symptomatic volunteers following challenge. There were no significant abnormalities in ECG, laboratory or spirometry parameters post-challenge in the influenza cohort.

## Discussion

The pH1N1/09 case definition was poorly predictive of influenza infection in this cohort of unimmunised healthy volunteers challenged with *P. falciparum* malaria or influenza A, in keeping with recent data on its performance in clinical practice^2^ as well as similar case-definitions in other settings.^10^ In our studies volunteers were treated as soon as significant symptoms or positive blood film developed, therefore only clinical features in the early phase of illness were addressed, and it remains possible that the power to distinguish between these infections clinically might be improved with a longer duration of illness. In addition the use of a non-pandemic challenge strain of influenza in this study may reduce generalisability of findings to pH1N1, which has higher pathogenicity in younger individuals^11^ presumably related to reduced pre-existing immunity^12^; yet all volunteers in our study had no detectable pre-existing immunity to the challenge strain, so in this context they might be considered more representative of a pandemic exposed population. Moreover, the pre-assessment probability of infection has a major impact on the PPV and NPV, so during a pandemic setting (where the prevalence of influenza would be significantly greater than malaria) the case definition would be expected to perform significantly better. However in agreement with our data several instances of misclassification of *P. falciparum*^4,5^ and other serious illnesses^9^ have been reported in the context of protocol-based case-definitions during the peak of community transmission during the pandemic, highlighting the limitations of such protocol or algorithm-based diagnosis.^5^ Telephone consultation is an important component of infectious disease practice,^13^ and telephone or internet-based triage has considerable practical advantages in pandemic settings, however the poor predictive capacity of the pandemic case-definition demonstrated here supports the revisions to the case definition to incorporate travel history.^3^

Only upper respiratory tract symptoms reliably distinguished malaria from influenza in our study. Whilst the nasal route of inoculation may have theoretically influenced the incidence of rhinorrhoea by delivering a higher multiplicity infection to the nasal mucosa, a recent influenza clinical case series identified cough as a presenting symptom in 92% of individuals with naturally-acquired proven influenza infection,^14^ suggesting that in the absence of upper respiratory symptoms, particularly cough,^15^ a presumptive diagnosis of influenza should be reconsidered.

Phase IIa controlled experimental infection studies play an important role in improving our understanding of the clinical features of infectious diseases.^16,17^ However for ethical reasons sample sizes for such studies are relatively small, thus our statistical power to assess differences in clinical features between malaria and influenza in the analysis presented here may have been reduced. In addition, influenza challenge protocols rarely result in 100% attack rates,^7^ as reflected in the 8/11 volunteers developing symptoms and the 5/8 symptomatic volunteers with laboratory-confirmed influenza following nasal challenge, which further impacted sample size. Nevertheless, several lines of evidence support the conclusion based on these data that clinical features alone poorly distinguish between these infections. Firstly there are no reliable clinical diagnostic criteria for influenza.^15^ Secondly both infections are frequently confused in febrile returning travellers, where influenza is a frequent diagnosis in travellers to malaria-endemic regions,^18–21^ as well as in individuals in malaria-endemic regions where a significant burden of undiagnosed influenza appears to exist in holoendemic transmission settings.^22^ Therefore keeping an open mind regarding the clinical cause of undifferentiated febrile illness, particularly in the absence of upper respiratory tract symptoms, remains important even during influenza pandemic settings.

## Conflict of interest

The authors declare no conflict of interest.

## Figures and Tables

**Table 1:** Demographic details of symptomatic controlled experimental infection volunteers.

Demographics	Malaria	Influenza	*P* value
No. of volunteers	11	8	–
Median age (IQR)	28.6 (22.5–35.4)	28 (23.0–37.0)	0.51^a^
Male/female	6/5	3/5	0.65^b^

a Mann–Whitney test.

b Fisher's exact test.

**Table 2:** Clinical features of symptomatic individuals following pathogen exposure.

Symptom	Malaria infected (*n* = 11)	Influenza exposed^a^ (*n* = 8)	*P* value^b^ (exposed)	Influenza infected^a^ (*n* = 5)	*P* value^b^ (infected)
Cough	0	5	0.005	4	0.003
Diarrhoea	2	0	0.49	0	1.0
Headache	10	6	0.55	4	1.0
Fever	8	2	0.18	2	0.55
Malaise	3	3	1.0	2	1.0
Myalgia/arthralgia	10	5	0.72	3	0.21
Nasal symptoms	1	7	0.001	5	0.001
Nausea/vomiting	3	2	1.0	1	1.0
Sore throat	0	3	0.058	3	0.018
Met case definition	8	3	0.18	3	1.0

a 8/11 influenza-exposed volunteers became symptomatic, 5/8 of whom developed viral culture-confirmed influenza infection.

b Analysis by two-tailed Fisher's exact test versus malaria-infected volunteers.
